# Correlation of HIV viral load, CD4 count and treatment response among HIV-positive patients in India

**DOI:** 10.6026/973206300220447

**Published:** 2026-01-31

**Authors:** Madhuri Gattani, Manish Purohit, Ashish Kumar Joshi

**Affiliations:** 1 Department of Microbiology, DR. Laxminarayan Pandey Government Medical College, Ratlam, Madhya Pradesh, India; 2 Department of Microbiology, MGM Medical College, Indore, Madhya Pradesh, India

**Keywords:** Human immunodeficiency virus (HIV), viral load, CD4 count, antiretroviral therapy, treatment response

## Abstract

The lack of comprehensive understanding regarding the correlation of HIV viral load, CD4 count and treatment response among HIV-positive individuals in India is a concern. Therefore, it is of interest to evaluate changes in CD4 cell count and HIV viral load following ART initiation, demonstrating a significant increase in CD4 count and decrease in viral load. It highlights the inverse correlation between viral load and CD4 count, emphasizing the complementary role of both in treatment monitoring. Routine viral load and CD4 testing are critical for optimal HIV care, particularly in resource-limited settings. Thus, we show enhances understanding of the correlation between HIV viral load and CD4 count in monitoring antiretroviral therapy (ART) effectiveness.

## Background:

Human immunodeficiency virus (HIV) infection remains a major global public health challenge despite significant advances in diagnosis, treatment and prevention strategies. HIV, a retrovirus belonging to the family retroviridae, primarily targets CD4^+^ tlymphocytes, leads to progressive immunodeficiency and increased susceptibility to opportunistic infections and malignancies [1]. If untreated, HIV infection advances to acquire immunodeficiency syndrome (aids), characterized by severe immune suppression and high mortality rates [2]. Globally, an estimated 39 million people were living with HIV by the end of 2022. Although the scale-up of antiretroviral therapy (art) has substantially reduced HIV-related morbidity and mortality, gaps persist in diagnosis, treatment coverage and viral suppression [3]. Approximately 86% of people living with HIV are aware of their status, 76% are receiving art and only 71% have achieved viral suppression, highlighting the need for continuous monitoring and optimization of treatment strategies [4]. India bears the second-largest burden of HIV worldwide, accounting for approximately 6.3% of the global population living with HIV. Despite notable progress under the national aids control programme (NACP), challenges such as delayed diagnosis, loss to follow-up, drug resistance, co-infections and treatment adherence continue to affect long-term outcomes. In India, nearly two-thirds of people living with HIV are on art, with viral suppression achieved in about 63% of treated individuals, emphasizing the importance of effective treatment monitoring tools [5]. The introduction of combination antiretroviral therapy, also referred to as highly active antiretroviral therapy (HAART), has transformed HIV infection from a fatal disease into a manageable chronic condition. Art suppresses viral replication, improves immune reconstitution, reduces HIV transmission and enhances overall quality of life. However, the effectiveness of art is influenced by several factors, including adherence to therapy, baseline immune status, viral characteristics, drug toxicity, emergence of resistance and socioeconomic determinants, particularly in resource-limited settings [6]. Monitoring treatment response in HIV-infected individuals is crucial for evaluating therapeutic success and guiding clinical decision-making. Plasma HIV viral load and CD4^+^ t-cell count are the two most important laboratory markers used to assess virological suppression and immunological recovery, respectively. Viral load reflects the level of active viral replication and is a strong predictor of disease progression and transmission risk, whereas CD4 count indicates the functional status of the immune system and risk of opportunistic infections [7]. Current national and international guidelines recommend routine viral load monitoring as the preferred method for assessing art efficacy, with CD4 count serving as an essential baseline and adjunct marker, particularly in advanced disease. A sustained reduction in viral load is generally associated with a gradual increase in CD4 count, though discordant responses may occur. Understanding the correlation between virological and immunological parameters is therefore essential for early identification of treatment failure, timely regimen modification and prevention of disease progression [8]. In resource-constrained settings, where access to frequent viral load testing may be limited, correlating CD4 cell count with viral load can provide valuable insights into treatment response and programmatic effectiveness. Evaluating these parameters in real-world clinical settings, such as tertiary care art centers, is particularly important to assess the impact of national HIV control strategies and to identify local determinants influencing treatment outcomes [9]. Therefore, it is of interest to evaluate the correlation between HIV viral load, CD4 cell count and treatment response among HIV-positive individuals attending the art centre of a tertiary care hospital in central India.

## Materials and Methods:

The present prospective observational study was conducted in the department of Microbiology, Mahatma Gandhi Memorial (M.G.M.) medical college in collaboration with the antiretroviral therapy (art) centre, M.Y. hospital, Indore, Madhya Pradesh.

The study was carried out over a period of one year, from 6 December 2022 to 5 December 2023.

## Inclusion criteria:

[1] Confirmed HIV-1 positive individuals

[2] Patients who had completed at least 6 months of art

[3] Patients attending the art centre for routine HIV-1 viral load and CD4 count testing

[4] Patients willing to participate and provide informed consent

## Exclusion criteria:

[1] Patients who were lost to follow-up during the study period

[2] Patients not willing to participate or not provide informed consent

## Sample size:

During the study period, a total of 110 HIV-1 seropositive patients fulfilling the eligibility criteria were enrolled in the study

## Data collection:

Detailed demographic and clinical data including: Age, gender, mode of HIV transmissions, presence of co-infections (tuberculosis, syphilis, hepatitis b, hepatitis c), baseline who clinical staging, baseline CD4 cell count were collected from art centre records. The enrolled participants were evaluated at 6 months of art and 12 months of art.

## Laboratory methods:

Venous blood samples were collected under aseptic precautions in EDTA vacutainers as per NACO guidelines for CD4 t-cell enumeration and HIV-1 plasma viral load estimation. CD4 cell counts were estimated using flow cytometry. CD4 enumeration was performed according to standard laboratory protocols recommended under the national aids control programme (NACP). CD4 count was expressed as cells/mm^3^ and used to assess immunological response to art HIV-1 viral load testing was performed using plasma HIV-1 RNA nucleic acid amplification tests (NAT).

Viral load values were categorized as:

[1] Undetectable (target not detected - TND)

[2] Suppressed viral load (<1000 copies/ml)

[3] Unsuppressed viral load (≥1000 copies/ml)

Interpretation of viral load results and patient management were done as per NACO guidelines

## Definition of treatment response:

Virological response was defined as plasma HIV-1 viral load <1000copies/ml after at least 6 months of art. Virological failure was defined as plasma viral load ≥1000 copies/ml after 6 months of art despite adherence counseling. Immunological response was defined as an increase in CD4 cell count of ≥50 cells/mm^3^ after 6 months of art. Immunological failure was considered when CD4 count persistently remained <100 cells/mm^3^ or failed to rise after art initiation.

## Ethical considerations:

The study was approved by the institutional ethics and scientific review committee, M.G.M. medical college, Indore.

## Statistical analysis:

Data were entered into Microsoft excel and analyzed using appropriate statistical methods. Categorical variables were expressed as frequency and percentage. Continuous variables were expressed as mean ± standard deviation. Associations between CD4 count, viral load and treatment response were analyzed using appropriate statistical tests. A p-value <0.05 was considered statistically significant.

## Results:

A total of 110 HIV- positive individuals on art were enrolled and analysed. There was a progressive and statistically significant rise in mean CD4 count from baseline to 12 months of art, indicating effective immunological recovery with continued therapy (Table 1). The proportion of patients with undetectable viral load increased significantly by 12 months, while those with virological failure (≥1000 copies/ml) declined (Table 2). There was a significant improvement in virological status over time, with an increase in the proportion of patients achieving undetectable viral load at 12 months and a corresponding decline in patients with high viral load levels (Table 3). At 6 months of art, most patients with undetectable viral load had higher CD4 counts, while those with higher viral load levels were more likely to have lower CD4 counts, suggesting an inverse relationship between viral load and CD4 count (Table 4). At 12 months of art, a greater proportion of patients with undetectable viral load demonstrated CD4 counts above 500 cells/mm^3^, further strengthen the inverse correlation between viral load and immunological status (Table 5). The majority of study participants achieved both virological suppression and immunological response at 12 months of art, while a small proportion exhibited discordant treatment responses (Table 6). Immunological non-response was more frequently observed among patients with tuberculosis co-infection, advanced who clinical stage, low baseline CD4 count and hepatitis b co-infection (Table 7). Virological non-suppression was more common among patients with tuberculosis; advanced who stage, low baseline CD4 count and syphilis co-infection, indicating an association between co-morbid conditions and poor virological response.

## Discussion:

The current study assessed the immunological and virological treatment response of HIV-positive patients receiving antiretroviral medication at a tertiary care center in central India, utilizing CD4 cell count and HIV viral load as monitoring indicators. A progressive and statistically significant increase in mean CD4 cell count was found from baseline to 6 months and then to 12 months of art, showing successful immunological reconstitution after medication commencement. This improvement is due to the reduction of viral replication and the eventual restoration of CD4 t-lymphocyte activity. Similar findings have been reported in longitudinal cohort studies, with the greatest CD4 increases occurring within the first year of art among patients who achieved prolonged viral suppression [10]. Virological surveillance revealed a significant decrease in HIV viral load with increasing duration of art. A significant majority of patients had undetectable viral loads after 6 months, with further improvement at 12 months. The decrease in the number of people with high viral load levels over time indicates excellent treatment response and adherence. Large art programmes have reported comparable virological outcomes after implementing routine viral load testing and tailored adherence assistance [11].

At 6 and 12 months of art, there was an inverse connection between HIV viral load and CD4 cell count. Individuals with undetectable viral loads had greater CD4 counts, whereas those with elevated viral loads had lower CD4 counts. This observation demonstrates that continuing viral replication has a deleterious impact on immune recovery, which is consistent with previous research that examined virological and immunological dynamics during art [12]. Although the majority of patients showed concordant virological and immunological responses, a small number had discordant results. Some people showed no immune response despite virological suppression, whereas others showed immunological improvement despite inadequate viral suppression. Such disparities in responses have previously been observed and may be impacted by baseline immunological status, severe disease stage, immune activation, or co-existing infections [13]. Further investigation found that immunological non-response was more common among patients with tuberculosis co-infection, advanced who clinical stage (iii/iv), low baseline CD4 count and hepatitis b co-infection. Tuberculosis continues to be a primary cause of immunological activation and delayed CD4 recovery in HIV-infected people, even after effective viral suppression is achieved [14]. Similarly, virological non-suppression was more likely in patients with tuberculosis, advanced illness stage, low baseline CD4 count and co-infection with syphilis. These data underscore the influence of co-infections and advanced HIV disease on treatment outcomes, emphasizing the necessity of early detection, quick art introduction and integrated co-morbidity management [15].

## Conclusion:

We show that antiretroviral medication leads to considerable virological suppression and long-term immunological recovery in HIV- positive patients attending a tertiary care art centre. A strong negative association was found between HIV viral load and CD4 cell count, highlighting their complementary significance in evaluating treatment response. Routine viral load testing, along with CD4 monitoring, remains critical for early diagnosis of treatment failure and optimisation of HIV care, especially in resource-constrained settings.

## Figures and Tables

**Table 1:** CD4 cell count at baseline, 6 months and 12 months of art among study patients

**Time interval**	**CD4 count (cells/mm^3^) (mean ± sd)**
Baseline (pre-art)	314.80 ± 244.84
6 months post-art	500.71 ± 293.68
12 months post-art	664.15 ± 336.67

**Table 2:** HIV viral load status at 6 and 12 months of art among study patients

**HIV viral load level range in copies/ml**	**Post art**	
	**At 6 months (n=110)**	**At 12 months (n=110)**
Tnd	79 (71.82%)	92 (83.64%)
<1000	17 (15.50%)	10 (9.10%)
1000-10,000	5 (4.55%)	6 (5.45%)
>10,000	9 (8.18%)	2 (1.82%)

**Table 3:** Relationship between viral load and CD4 count at 6 months of art

**Viral load group after 6 months of art**	**N=110**	**CD4 count after 6 months of art**		
		**<200**	**200-500**	**>500**
Tnd	79	5 (4.5%)	41 (37.3%)	33 (30%)
<1000	17	2 (1.8%)	10 (9.1%)	5 (4.5%)
1000-10,000	5	1 (0.9%)	3 (2.7%)	1 (0.9%)
>10,000	9	3 (2.7%)	4 (3.6%)	2 (1.8%)

**Table 4:** Relationship between viral load and CD4 count at 12 months of art

**Viral load level after 12 months of art**	**N=110**	**CD4 count after 12 months of art**		
		**<200**	**200-500**	**>500**
Tnd	92	4 (3.6%)	22 (20.0%)	66 (60%)
<1000	10	1(0.9%)	4 (3.6%)	5 (4.5%)
1000-10,000	6	0 (0.0%)	3 (2.7%)	3 (2.7%)
>10,000	2	1 (0.9%)	1 (0.9%)	0 (0.0%)

**Table 5:** Overall treatment response at 12 months of art

**Response**	**Immunological response**	**Immunological nonresponse**
Virologic suppression	96 (87.27%)	6 (5.4%)
Virologic non suppression	7 (6.3%)	1 (0.90%)

**Table 6:** Factors associated with immunological status after 12 months of art (n=110)

**Characteristics**	**Immunologically**	**Immunologically responsive (n=103)**
	**Non-responsive (n=7)**	
Co-infection with tuberculosis (n=24 )	4 (16.66%)	20 (83.33%)
Who staging III & IV (n=29)	4 (13.79%)	25 (86.20%)
HIV positivity in their spouse (n=41 )	6(14.63%)	35 (85.36%)
Low baseline CD4 count (n=40)	3 (7.5%)	37 (92.5%)
Co-infection with hepatitis b (n=5)	1 (20%)	4 (80%)
Male (n=81)	5 (6.17%)	76 (93.82%)
Female (n=28)	2 (7.14%)	26 (92.85%)

**Table 7:** Factors associated with virological status after 12 months of art (n=110)

**Characteristics**	**Virological Non-suppression (n=8)**	**Virological suppression (n=102)**
Co-infection with tuberculosis (n=24 )	3 (12.5%)	21 (87.5%)
Who staging III & IV (n=29)	4 (13.79%)	25 (86.20%)
Spouse positive (n=41)	4 (9.75%)	37 (90.24%)
Low baseline CD4 count <200 (n=40)	4 (10%)	36 (90%)
Co-infection with syphilis (n=8)	1 (12.5%)	7 (6.8%)
Male (n=81)	5 (6.17%)	76 (93.82%)
Female (n=28)	3 (10.71%)	25 (89.28%)

## References

[1] Soni T, Oral Sphere J (2025). Dent. Health Sci..

[2] Balasubramaniam M (2019). J Life Sci (Westlake Village)..

[3] Du S (2025). BMC Infect Dis..

[4] Kyere GA (2024). BMC Infect Dis..

[5] Tanwar S (2016). J Virus Erad..

[6] Mahungu TW (2009). Clin Med (Lond)..

[7] Vajpayee M (2011). Indian J Med Res..

[8] Rowley CF (2014). Clin Infect Dis..

[9] Manoto SL (2018). Medicina..

[10] Patel V (2022). IP Indian J Immunol Respir Med..

[11] Gaifer Z, Boulassel MR (2020). J Int Assoc Provid AIDS Care..

[12] Abadi T (2024). Eur J Med Res..

[13] Saison J (2014). Clin Exp Immunol..

[14] Sharma K (2025). Pathogens..

[15] Getaneh T (2022). J Clin Tuberc Other Mycobact Dis..

